# Substandard housing and the risk of COVID-19 infection and disease severity: A retrospective cohort study

**DOI:** 10.1016/j.ssmph.2024.101629

**Published:** 2024-02-13

**Authors:** Katharine Robb, Rowana Ahmed, John Wong, Elissa Ladd, Jorrit de Jong

**Affiliations:** a Bloomberg Center for Cities, Harvard Kennedy School, Cambridge, MA, USA; b School of Nursing, MGH Institute of Health Professions, Boston, MA, USA

**Keywords:** Housing, COVID-19, Housing code violations, Lockdown

## Abstract

In this study we examine associations between substandard housing and the risk of COVID-19 infection and severity during the first year of the pandemic by linking individual-level housing and clinical datasets. Residents of Chelsea, Massachusetts who were tested for COVID-19 at any Mass General Brigham testing site and who lived at a property that had received a city housing inspection were included (N = 2873). Chelsea is a densely populated city with a high prevalence of substandard housing. Inspected properties with housing code violations were considered substandard; inspected properties without violations were considered adequate. COVID-19 infection was defined as any positive PCR test, and severe disease defined as hospitalization with COVID-19. We used a propensity score design to match individuals on variables including age, race, sex, and income. In the severity model, we also matched on ten comorbidities. We estimated the risk of COVID-19 infection and severity associated with substandard housing using Cox Proportional Hazards models for lockdown, the first phase of reopening, and the full study period. In our sample, 32% (919/2873) of individuals tested positive for COVID-19 and 5.9% (135/2297) had severe disease. During lockdown, substandard housing was associated with a 48% increased risk of COVID-19 infection (95%CI 1.1–2.0, p = 0.006). Through Phase 1 reopening, substandard housing was associated with a 39% increased infection risk (95%CI 1.1–1.8, p = 0.020). The difference in risk attenuated over the full study period. There was no difference in severe disease risk between the two groups. The increased risk, observed only during lockdown and early reopening – when residents were most exposed to their housing – strengthens claims that substandard housing conveys higher infection risk. The results demonstrate the value of combining cross-sector datasets. Existing city housing data can be leveraged 1) to identify and prioritize high-risk areas for future pandemic response, and 2) for longer-term housing solutions.

## Highlights

• Living in substandard housing is linked to an increased risk of COVID-19 infection.
• The increased infection risk is only observed during Lockdown and early reopening.
• Substandard housing is not linked to greater risk of severe COVID-19 disease.
• Leaders can leverage city housing data for pandemic response and longer-term solutions.

## 1 Introduction

Housing is a powerful determinant of health, impacting social relationships, environmental exposures, safety, and a range of other outcomes. Living in substandard housing can increase the risk of infectious disease, mental illness, chronic disease, and physical injury (Adamkiewicz et al., 2014; Jacobs, 2011; Krieger & Higgins, 2002). Amid guidance to stay at home during the COVID-19 pandemic, safe housing was even more critical.

Substandard housing has been shown to be a risk factor for respiratory infections through several mechanisms (Krieger & Higgins, 2002). Poor ventilation, overcrowding, and dampness create favorable environments for microorganisms, molds, and pests, all of which can play a role in respiratory disease transmission and susceptibility (Brunekreef et al., 1989; Coulburn & Miller, 2022; Dales et al., 1991; Hynes et al., 2004; Peat et al., 1998). Overcrowding further increases the opportunity for person-to-person transmission. Unsafe or unsanitary conditions within homes may preclude extended periods of time at home which may also increase the risk for respiratory infection. For example, people living in homes without adequate protection from the elements or with water leaks, insect or animal infestations, or other hazards may seek refuge in public spaces or in the homes of others, increasing infection risk (Ahmad et al., 2020). Given the associations between substandard housing and chronic diseases like asthma and diabetes – conditions like overcrowding, poor ventilation, mold, and infestations not only may increase exposure to infectious disease but may also make people more susceptible to infection and severe disease (Benfer et al., 2021; Mehdipanah, 2020).

Exposure to substandard housing– defined as overcrowding, rent greater than 50% of income, or incomplete kitchen or bathroom facilities – has been independently associated with increased incidence and mortality from COVID-19 when examined at the county-level in the U.S. (Ahmad et al., 2020). The association persisted after controlling for county-level demographics, income, comorbidities, and access to healthcare. Several empirical and numerous editorial pieces have posited that inequalities in housing conditions contributed to inequalities in COVID-19 infection and severity (Bambra et al., 2020; Barry, 2020; Harlem, 2020; Logan, 2020a; Mehdipanah, 2020). Few studies, however, have explored links between social determinants of health and COVID-19 by analyzing data at the individual level, relying instead on area-level socioeconomic factors (Lee et al., 2022). While useful in generating hypotheses and exploring population-level trends, ecological studies are limited in that the relationships observed at the population level may not hold true at the individual level. To our knowledge, there have been no rigorous studies of individual-level housing exposures and the risk of COVID-19. If substantiated, the risk posed by substandard housing for COVID-19 infection and severity is both plausible and important for policy and programs.

### 1.1 Objective

In this study, we estimate the impact of substandard housing on the risk of COVID-19 infection (Research Question A) and COVID-19 disease severity (Research Question B) in Chelsea, Massachusetts, USA during the first year of the pandemic. To do this, we combined two data sources from across sectors – city housing data and health system data – to yield new insights into the impact of housing conditions on COVID-19 infection risk and disease severity. We also sought to examine how well preexisting markers of disadvantage held in administrative city datasets (e.g., housing code violations) map onto disease risk to help city and public health leaders leverage these data to respond more proactively and effectively.

### 1.2 Context

#### 1.2.1 Setting

Chelsea is a densely populated, demographically diverse city near Boston with a population estimated at 40,000 (US Census, 2020). However, informal estimates of the population are as high as 75,000 due to the city’s high proportion of undocumented immigrants who are less likely to participate in census counts (The Boston Globe, 2020). On just 2 square miles of land, the informal estimates make Chelsea one of the most densely population cities in the country (Kowalczyk & Greenberg, 2020). Chelsea embodies the nation’s affordable housing crisis, and several lines of evidence suggest this amplified infection during the pandemic. High rents and low wages force many into unsafe and unstable housing conditions (Ambrosino, 2017). Residents double- or triple-up with other families or live in illegal “conversion apartments” (e.g., basements, closets, or porches that often lack adequate heat, ventilation, and sanitation facilities) to avoid homelessness and stay close to jobs. The 2020 census showed that 72% of Chelsea residents were renters and 23% lived in poverty. Two-thirds of residents are Hispanic/Latino (66%) and almost half are foreign-born (47%). In 2019, the city’s top employment categories were food service (13%), cleaning and maintenance (9%), administrative support (8%), sales (8%), and production occupations (7%) (Data USA, 2020). Many of these job types were either deemed essential during the pandemic or were not compatible with remote work. Unemployment in Chelsea skyrocketed to 21.2% in April 2020, up from 3.2% in February 2020 (Mass.gov, 2023). Rates of COVID-19 in Chelsea were six times the state average in 2020 (Barry, 2020). In April 2020, just two months into the COVID-19 pandemic, a seroprevalence study conducted on a convenience sample of 200 asymptomatic residents of Chelsea who walked past a testing site found that 31.5% had antibodies suggestive of recent COVID-19 infection (Naranbhai et al., 2020).

#### 1.2.2 COVID-19 timeline in Massachusetts

On March 10th, 2020, the first evidence of COVID-19 community transmission in Massachusetts was recorded. That same day, the governor declared a state of emergency. On March 15th schools and many businesses closed. Dining in restaurants and gatherings of more than 25 people were banned. On March 23rd a stay-at-home advisory was issued, and nonessential businesses were ordered closed.

By mid-May 2020, hospitalizations declined and phased reopening began. Phase 1 (May 18th-June 8th) allowed only manufacturing facilities, construction sites, and places of worship to reopen. Reopening continued in phases until the state of emergency was lifted in June 2021 (Mass.gov, 2020).

In this study, we focus on the first year of the pandemic – from March 1st, 2020, through February 28th, 2021. We divide the year into three overlapping periods which we refer to as 1) Lockdown – March 1st through May 17th, 2) Lockdown plus Phase 1 Reopening – March 1st through June 7th, and 3) Year 1 of the COVID-19 Pandemic– March 1st, 2020, through February 28th, 2021. During the study period, a vaccine was not available for the general public. The primary mitigation strategy for COVID-19 was to stay at home and avoid close proximity with others, poorly ventilated spaces, and contaminated surfaces.

## 2 Methods

### 2.1 Clinical dataset

Sociodemographic and health data were extracted from Mass General Brigham’s (MGB, formerly known as Partners Healthcare) Research Patient Data Registry, a centralized clinical data registry for the MGB healthcare system. Only patients with a home address in Chelsea and who had a COVID-19 test (positive or negative by PCR) between March 2020 and March 2021 were included. Variables extracted included patients' address, demographic characteristics, insurance type, COVID-19 testing history, and assignment of a primary care provider (PCP). Most patients also had medical record data in addition to the testing variables listed above. For patients with a medical record, we also extracted chronic medical conditions and outpatient and inpatient visit history.

Race/ethnicity and primary language variables were based on fixed response categories within the health record. An income variable indicating whether a patient was above or below 300% the Federal Poverty Level was inferred from the patients' health insurance type. To qualify for state subsidized health insurance, 300% of poverty was the income maximum. Below 300% of poverty, it was not possibly to reliably determine income level based on health insurance type (MA Medicaid Policy Institute, 2022). Chronic health conditions were identified from ICD-9 and ICD-10 codes found in the patients’ encounter history during the 10-year period preceding March 2020. Target ICD-9 and ICD-10 codes were categorized into the following disease groups: Asthma, Chronic Obstructive Pulmonary Disease (COPD), Cancer, Diabetes, Heart Disease, Hypertension, Kidney Disease, Lung Disease, Obesity, and Prediabetes, as these comorbidities were used to prioritize high risk groups for eventual COVID-19 vaccination (Mass.gov, 2021). Patients without medical records and those with medical records only after the date of their first COVID-19 test were excluded from the severe disease model but included in the infection risk model. While there is substantial evidence of underlying health conditions increasing the risk of severe COVID-19 disease, high-quality evidence of the impact of health conditions on infection risk is lacking (Pijls et al., 2021).

MGB is the primary healthcare system serving Chelsea residents (CMS, 2020; Spiro et al., 2012). During the study period, at-home tests were not available and testing at healthcare facilities was free of charge. Nonetheless, there were undoubtably Chelsea residents who had COVID-19 and did not seek testing or sought testing and care through different healthcare systems – these residents are not captured in our study.

### 2.2 Housing dataset

The City of Chelsea runs a proactive housing inspection program whereby rental properties are inspected according to a schedule regardless of whether there was a housing complaint. The result of a housing inspection can be no violations or one or more violations. Housing code violations are issued when properties fail to meet minimum health and safety standards. We compiled a dataset that included the addresses of all residential properties in Chelsea. Using data from Chelsea City Hall, we linked city administrative data and data on housing inspection associated with each address. The details of this dataset have been described previously (Robb, Diaz Amigo, et al., 2021).

### 2.3 Merged dataset

We merged patient addresses (N = 6637 patients) with addresses in the housing dataset (N = 5963 properties). While the housing dataset contained data on all residential properties in Chelsea, not all patients resided at a residential property (e.g., nursing homes, Veterans’ housing). Further, due to clerical errors and discrepancies between administrative and postal addresses, not all addresses in the patient dataset matched to an address in the housing dataset. For these reasons, 1826 patients were excluded. We also excluded properties that had not received a proactive housing inspection because the code violation status of these residences was unknown (N = 2456). The remaining 2873 inspected properties where patients tested for COVID-19 resided were used in the analysis for Research Question A. Research Question B utilized the patients from Research Question A that also had medical records prior to their first COVID-19 test date (N = 2297). Comparable rates of medical record missingness were observed in both residents of adequate housing and substandard housing.

### 2.4 Exposure

Substandard housing was defined as one or more housing code violations issued by the City of Chelsea between 2015 and 2019. The state sanitary code contains 38 violation types (MA Department of Public Health, 2007). Drawing from the housing dataset, the five most common violations in order of frequency were: owner failing to maintain structural elements; lack of smoke or carbon monoxide detectors; overcrowding; inadequate kitchen facilities; and infestation of insects or rodents. When found, violations must be resolved within a set timeframe or a fine is issued. However, many violations are temporarily or inadequately resolved (Stacy et al., 2018). This phenomenon was described by Chelsea housing inspectors (Robb, Marcoux, & de Jong, 2021). In Boston, in neighborhoods with the fewest White residents compared to the most White residents, a study found that there was a 54% lower probability of a repair taking place in the context of a housing code violation, even when adjusting for important covariates (Lemire et al., 2022). The density of housing code violations was associated with population-level morbidity independent of poverty in a study in Cincinnati, Ohio (Beck et al., 2014). In the present study, we use living in a home with a history of housing code violations as a proxy for living in substandard housing conditions.

Still, exposure misclassification for some portion of our sample is likely. Conditions within homes could have changed (worsened or improved) before and during the study period. Further, a portion of the study population likely moved residences, yet we assume the address on file in March 2021 is the address where the patient lived during and prior to the pandemic. While rising rents and evictions force many Chelsea residents to move frequently (Logan, 2020b), during the study period moving was likely less common due to the eviction and foreclosure moratorium in place from April 2020 through August 2021 (Connolly & Honan, 2020). Additionally, for properties that had one parcel ID associated with multiple units, it was not possible to distinguish to which unit on the property a violation accrued; violations were assigned at the parcel-ID level. Further, while inspectors are trained to be objective, inspection is subject to human error and subjectivity (Bartram, 2022). Lastly, we did not include analysis of specific code violations due to small samples sizes and coarse code violation definitions. These factors may bias the analysis away from detecting an effect. Nonetheless, this method for assessing housing quality is advantageous because 1) in-person evaluation of housing conditions was not feasible during pandemic restrictions and resource prohibitive at a city level, and 2) the method takes advantage of data that exists in many cities such that the approach could be replicated.

### 2.5 Outcomes

#### 2.5.1 Risk of infection (Research Question A)

Infection with COVID-19 was defined as a positive PCR test during the study period. If multiple positive tests were documented, only the date of the first positive test was used.

#### 2.5.2 Risk of severe disease (Research Question B)

Severe COVID-19 disease was defined as hospitalization during the study period with a diagnosis of COVID-19. If multiple hospitalizations occurred, the date of the first hospitalization with COVID-19 was used. At the start of the pandemic, the COVID-19 ICD-10 code (U07.1) did not exist. Interim ICD-10 codes recommended by the CDC indicating exposure to the virus in the presence of related symptoms were used from March 1st - April 1st, 2020, to identify patients who were hospitalized with COVID-19 (CDC, 2020).

### 2.6 Analytical approach

#### 2.6.1 Matching

We used propensity score-matching to examine the independent effect of substandard housing on the risk of testing positive for COVID-19 and of developing severe disease. Propensity scores were calculated for each patient (N = 2873) using a full match with a probit distance function (Hansen, 2004). Each patient in both exposed (substandard housing) and unexposed (adequate housing) was assigned to a subclass. Each subclass contained one or more exposed and one or more unexposed patient. Weights were computed based on subclass membership, and these weights functioned as propensity score weights to estimate a weighted treatment effect. Variables used in matching were sex (male/female), race (White Non-Hispanic/Other), insurance status (yes/no), PCP status (yes/no), below 300% of poverty (yes/no), and the year the home was built. Exact matching was used for the patient’s age, binned into 20-year increments, and primary language (English/other), as these variables were found to be statistically significant factors for infection risk in exploratory modeling. Language was also a more reliable variable to capture key information to systematic barriers to accessing healthcare as the race variable collapsed most patients into an “Other” category.

In the COVID-19 severity model (Research Question B), a separate matched cohort was created due to the inclusion of the ten comorbidity statuses in the variables used for matching. A full match with a probit distance function was used for this cohort as well, with eight individuals from the exposed group being discarded due to lack of appropriate controls for matching in the dataset. The final matched dataset for Research Question B included 2297 patients.

#### 2.6.2 Time to event analysis

In each of the two matched cohorts, unadjusted Cox regression models were used to estimate hazard ratios – attributable to substandard housing – for testing positive for COVID-19 and severe disease across the three overlapping timeframes: Lockdown, Lockdown and Phase 1 Reopening, and Year 1 of the COVID-19 pandemic. Clustered errors at the matched subclass level were used to account for potential lack of independence between patients. Hazard ratios are an estimate of relative risk, which is the ratio of the probability of the event occurring in the exposed group versus the unexposed group (Spruance et al., 2004). In this paper, we refer to risk, as opposed to hazard, for ease in interpretation. By using overlapping timeframes, we were able to analyze the same initial population in subsequently longer timeframes without needing to use different eligible populations for each timeframe.

## 3 Results

During the first year of the pandemic, 32% (919/2873) of the study population tested positive for COVID-19 and 6% (135/2297) had severe disease. Over half, 57% (1624/2873) lived in substandard housing. Among those in substandard housing, 35% tested positive for COVID-19. Significantly fewer residents of adequate housing tested positive for COVID-19 (29%, *p* = 0.002) (Table 1). There was no difference in the percentage who developed severe disease between those in substandard versus adequate housing (*p* = 0.889).

**Table 1:** Characteristics of Study Population Exposed to Adequate versus Substandard Housing, Variables used in Matching (Pre-Match) and Outcome Variables.

Research Question A (N = 2873): Risk of Infection				
Variable	Total N (%)	Adequate Housing N (%)	Substandard Housing N (%)	*p*-value
Female	1610 (56.0)	726 (58.1)	884 (54.4)	0.048
Age (mean, sd)	39.9 (21.3)	42.8 (22.4)	37.7 (20.1)	<0.001
Non-White Race	2245 (78.1)	879 (70.4)	1366 (84.1)	<0.001
English as Primary Language	1128 (39.3)	620 (49.6)	508 (31.3)	<0.001
Income below 300% of Poverty	2182 (76.0)	899 (72.0)	1283 (79.0)	<0.001
Health Insurance	2523 (87.8)	1113 (89.1)	1410 (86.8)	0.063
Has Primary Care Provider	2227 (77.5)	980 (78.5)	1247 (76.8)	0.286
Year Home Was Built (mean, sd)	1924 (35)	1932 (40)	1918 (29)	<0.001
Outcome Variable: Covid Positive	919 (32.4)	363 (29.4)	556 (34.7)	0.002
*Totals*	*2873*	*1249 (43.5)*	*1624 (56.5)*	
**Research Question B (N = 2297): Risk of Severe Disease**				
**Variable**	**Total N (%)**	**Adequate Housing N (%)**	**Substandard Housing N (%)**	***p*-value**
Female	1363 (59.3)	610 (61.1)	753 (58.0)	0.152
Age (mean, sd)	40.7 (21.3)	44.1 (23.04)	38.2 (20.7)	<0.001
Non-White Race	1783 (77.6)	692 (69.3)	1091 (84.1)	<0.001
English as Primary Language	980 (42.7)	530 (53.1)	450 (34.7)	<0.001
Income below 300% of Poverty	1735 (75.5)	715 (71.6)	1020 (78.6)	<0.001
Health Insurance	2160 (94.0)	951 (95.2)	1209 (93.1)	0.048
Has Primary Care Provider	1994 (86.8)	881 (88.2)	1113 (85.7)	0.307
Year Home Was Built (mean, sd)	1925 (35)	1933 (40)	1919 (30)	<0.001
Asthma	535 (23.2)	271 (27.1)	264 (20.3)	<0.001
Cancer	238 (10.4)	123 (12.3)	115 (8.9)	0.009
COPD	68 (3.0)	50 (5.0)	18 (1.4)	<0.001
Diabetes	441 (19.1)	215 (21.5)	226 (17.4)	0.15
Heart Disease	768 (33.3)	375 (37.5)	393 (30.3)	<0.001
Hypertension	698 (30.4)	364 (36.4)	334 (25.7)	<0.001
Kidney Disease	293 (12.8)	157 (15.7)	136 (10.5)	<0.001
Lung Disease	167 (7.3)	101 (10.1)	66 (5.1)	<0.001
Obesity	1077 (46.9)	471 (47.1)	606 (46.7)	0.86
Prediabetes	111 (4.8)	50 (5.0)	61 (4.7)	0.81
Covid Positive	725 (31.6)	286 (28.6)	439 (33.8)	0.009
Outcome Variable: Covid Hospitalized	135 (5.9)	60 (6.0)	75 (5.8)	0.889
*Totals*	*2297*	*999 (43.5)*	*1298 (56.5)*	

Note: sd = standard deviation. *P*-values result from Chi-squared test for difference in proportions or *t*-tests for difference in group means (Age and Year Built).

**Fig. 1:**
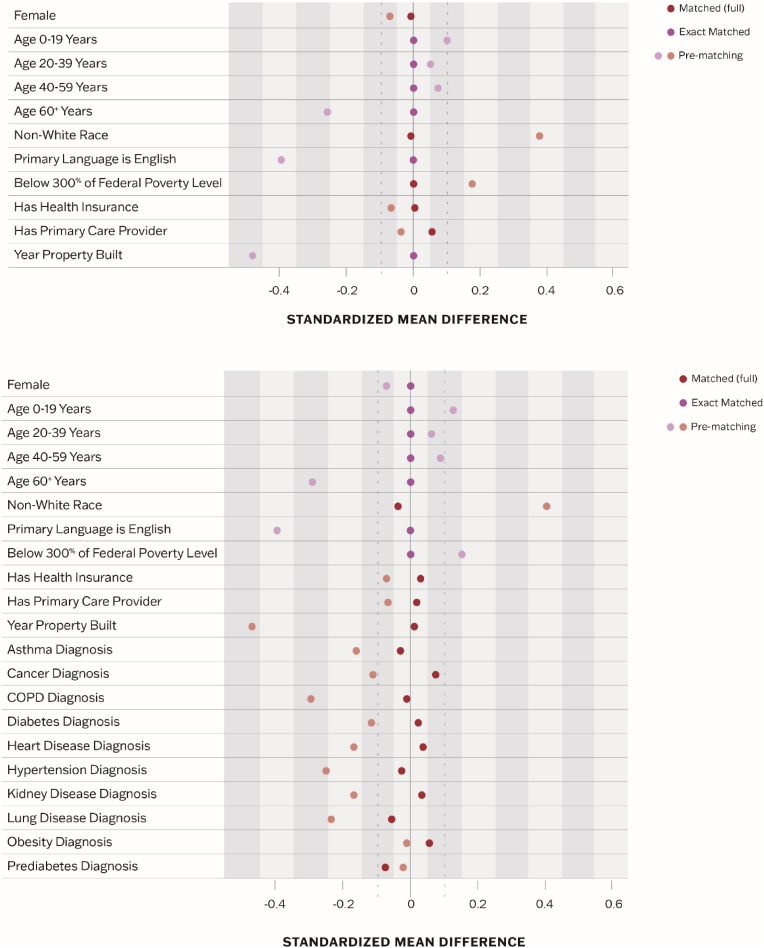
Covariate Balance for Variables Used in Matching (Research Question A, top, and B, bottom).

Before matching, individuals in substandard housing were more likely to be female, younger, and of a non-White race, and to not speak English as a primary language, live below 300% of poverty, and live in older homes compared to those in adequate housing (Table 1). Those in substandard housing were less likely to be diagnosed with chronic conditions associated with increased risk of severe disease from COVID-19. There was no difference between the two groups with regard to the percentage of the population with health insurance (89.1% versus 86.8%, *p* = 0.063) or a primary care provider (78.5% versus 76.8%, *p* = 0.286). After matching, standardized mean differences of <0.1 for the variables used in matching indicated good balance between the exposed and unexposed groups (Fig. 1).

We also assessed several variables not used in matching to better describe our study population (Supplementary Material). Notably, there was no difference between the two groups in terms of the proportion living at the same property as another person in the sample who tested positive for COVID-19. More COVID tests were received by people living in adequate housing compared to substandard housing. Lastly, there was no difference in measured COVID-19 severity outcomes between patients in adequate versus substandard housing: emergency department visits, intensive care unit admissions, or deaths.

**Fig. 2:**
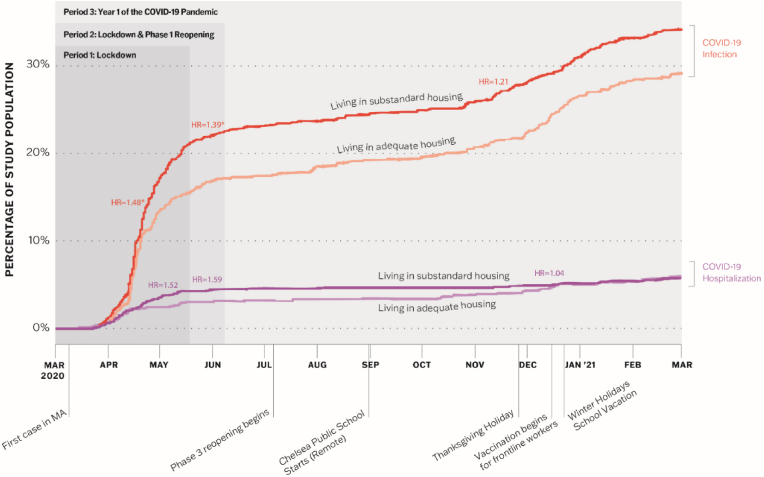
Cumulative proportion of study population testing positive for COVID-19 or hospitalized with COVID-19 living in adequate versus substandard housing.

In Fig. 2, the slope of the lines shows the rate at which the study populations living in adequate versus substandard housing developed COVID-19 infection (in red, Research Question A) and severe disease (in purple, Research Question B) over the first year of the pandemic. There was no difference in the risk of infection between the two groups when examining the full year. However, there was a significantly higher risk of infection for those living in substandard housing during Lockdown and Lockdown plus Phase 1 Reopening. This difference is visible in Fig. 2; the percentage of the population with a positive COVID-19 test increases more rapidly for those in substandard housing compared to adequate housing during the first two time periods. There were few new cases during the summer of 2020 regardless of housing conditions. Both groups experienced equivalent increases in infection in the fall of 2020 and winter of 2021. This is visible in the parallel trends in new infections in both groups beginning in late spring 2020. In examining disease severity, hospitalizations rates were low but climbed more precipitously in spring 2020 for both groups.

After matching, we found that living in substandard housing during Lockdown was associated with a 48% increased risk of COVID-19 infection (HR 1.48, 95% CI 1.1–2.0, *p* = 0.006) (Table 2). Through Lockdown and Phase 1 reopening, living in substandard housing was associated with a 39% increased infection risk (HR 1.39, 95% CI 1.1–1.8, *p* = 0.020) compared to adequate housing. The difference in risk was attenuated over the full study period. There was no difference in the risk of severe disease between people living in substandard versus adequate housing during any of the study time periods (Table 2).

**Table 2:** Living in substandard housing and the risk of COVID-19 infection and severe disease across three time periods.

	Hazard Ratio	95% CI	*p*-value
**Risk of Infection (Research Question A)**			
Lockdown	1.48	1.12–1.97	0.006
Lockdown & Phase 1 Reopening	1.39	1.10–1.80	0.020
Year 1 of Covid-19 Pandemic	1.21	0.95–1.50	0.131
**Risk of Severe Disease (Research Question B)**			
Lockdown	1.52	0.86–2.67	0.142
Lockdown & Phase 1 Reopening	1.59	0.92–2.76	0.099
Year 1 of Covid-19 Pandemic	1.04	0.65–1.65	0.873

Note: CI = confidence interval; *p*-values result from Cox Proportional Hazard models testing difference in time to event (infection or severe disease) between patients in substandard versus adequate housing.

## 4 Discussion

In this propensity-matched retrospective cohort study with a high prevalence of substandard housing and high rates of COVID-19, we found that patients living in substandard housing had an increased risk of developing COVID-19 infection. The higher risk was only observed during the periods of greatest exposure to conditions inside homes – Lockdown (48% greater risk) and Lockdown & Phase 1 Reopening (39% greater risk). The findings suggest that substandard housing is associated with higher COVID-19 infection risk.

Possible causal drivers of increased infection risk include housing code violations related to poor ventilation, overcrowding, and physical hazards (e.g.*,* holes in floor, broken staircases). These conditions can place individuals at greater risk of infection by decreasing circulation of clean air within a home (Poulin et al., 2022), and limiting control over risk mitigation (e.g., inadequate access to/control over kitchen or bathroom facilities; need for greater time spent in public spaces) (Kamis et al., 2021; Soltan et al., 2021).

The lack of difference in infection risk once stay-at-home advisories were lifted and people returned to – albeit limited – work and social life suggests that other factors and behaviors become more important to infection risk when exposure to housing decreased. With warmer weather in Summer 2020, it is also likely that time indoors decreased compared to Spring 2020. The rise in infections in Fall 2022 and Winter 2023 coincides with other possible drivers of infection risk such as increased social gatherings (Thanksgiving and winter holidays) and more people going to work (unemployment was 9.7% by December 2020) (Mass.gov, 2023).

We did not find evidence of housing conditions influencing the risk of severe COVID-19 disease (as measured by hospitalization). While population-level studies have found that disease severity was greater in areas with poorer housing (Ahmad et al., 2020), this was not the case in our individual-level study. Housing may increase the risk of becoming infected through the mechanisms described above. However, other characteristics such as a person’s age and comorbidities likely are more important in dictating disease severity (Pijls et al., 2021; Vaughan et al., 2021). Still, operationalizing severe COVID-19 disease as hospitalization with COVID-19 misses the portion of people who would meet the criteria for hospitalization but did not seek medical care. While we were able to match on some factors that may influence healthcare seeking behavior (insurance status, assignment of a primary care provider) other unmeasured confounders remain such as health system mistrust due to experiences of racial discrimination or concerns regarding immigration status (Zhang et al., 2022). Additionally, during COVID-19 peaks, widely publicized warnings of hospitals at surge capacity and the use of “field hospitals” may have deterred patients from seeking care (McCluskey, 2020). Given these limitations, and the magnitude of the hazard ratios in the disease severity models for Lockdown and Phase 1 Reopening, it is possible larger studies, studies with a different operationalization of severe disease, or studies with a different demographic makeup may find an association between housing conditions and COVID-19 severity.

Nonetheless, poor housing conditions are intimately intertwined with risk factors for COVID-19 infection *and* severe disease such as race, poverty, and health conditions (Badalov et al., 2022; Karmakar et al., 2021; Mackey et al., 2021). While we were able to control for many of these variables in our analysis, important variables related to housing conditions and COVID-19 risk were not available – such as type of employment and more granular information on income. People living in substandard housing may have been more likely to work low-wage jobs – the kind deemed essential during the pandemic and that carried a higher exposure risk (Morath & Feintzeig, 2020). It is possible that the risk we attribute to substandard housing may be due to unmeasured confounders, such as frontline worker status. Still, some of the variation in frontline worker status in our sample may be captured through matching on whether or not the patient had health insurance and was above or below 300% of poverty.

Despite limitations, this study has several strengths over previous studies on housing and COVID-19. Many health, demographic, and socio-economic databases aggregate data at the ZIP code or county level. Population-level studies, while effective for hypothesis generation and exploring population-level patterns, come with the constraint that the relationships identified at the population level might not be applicable to individual members within that group. Individual-level studies can adjust for individual-level confounders. In this study, we leverage an underutilized source of individual-level data by merging existing and often publicly available city administrative data with health data. Further, we used propensity score matching to balance observed covariates between individuals in exposed and unexposed groups, thereby reducing the impact of confounding.

Over the course of the pandemic, numerous studies were published on social determinants of health and COVID-19 infection and severity. COVID-19 is far from the first pandemic in which the role of socioeconomic factors has been described. Literature dating from the 19th century details the effect of socioeconomic inequality and disease incidence, including the risks posed by poor housing conditions (Koch, 2022; Krieger & Higgins, 2002). Past pandemics have shaped the housing codes in place today. These codes, many of which originated over a century ago, mandated minimum conditions for habitability including adequate ventilation and sanitary conditions to reduce the spread of infectious disease. As a result of housing codes and many other advances, homes became safer and infectious disease rates plummeted over the 20th century (Björkman, 2012; Krieger & Higgins, 2002). Yet, the COVID-19 pandemic exposed the tenement-like conditions that many thought had vanished at the end of the 19th century. The pandemic revealed a need for both better crisis response in addressing social determinants of health and for better data to understand how housing intersects with disease risk to inform long-term policy solutions. A more problem-driven approach to pandemic preparedness and housing requires intentional cross-sector efforts, novel approaches, and data analytic capacity (Mayne et al., 2020).

### 4.1 Implications and recommendations

#### 4.1.1 Pandemic preparedness recommendations

1. Bring together cross-sector datasets to yield new insights and solutions when faced with novel health crises. For example, population health divisions of medical centers or accountable care organizations (ACOs) can implement proactive infectious disease risk assessment and intervention (Amon et al., 2022) in concert with local authorities who have relevant data and resources related to housing conditions and other social determinants of health.
2. Prioritize areas with poor quality housing for risk mitigation such as A) increased availability of mobile bathrooms and cleaning supplies, B) engineering controls to improve ventilation and reduce airborne pathogens, C) deployment of healthcare practitioners to assess living conditions and respond to health risks, and D) setting up non-congregate shelters for people to safely isolate.

#### 4.1.2 Longer-term solutions to improve housing and health

1. Use available city data to identify and prioritize properties with the highest need for proactive inspections and inspections that integrate compliance with compliance assistance. Housing code enforcement matched with support services can help address underlying causes of poor housing conditions such as poverty and mental illness leading to more sustained improvements. See Robb et al. for examples (2021a, 2021b).
2. Leverage innovative strategies within healthcare to address social determinants of health. For example, some medical systems and ACOs provide social services through partnerships with local agencies. Examples include temporary housing and addressing environmental triggers such as mold (Beckman et al., 2021; Wortman et al., 2020).
3. Work toward longer-term solutions to increase the availability of safe housing. Faced with high rental costs, many low-income families withstand poor housing quality to make ends meet (Dunn, 2000). Increasing the availability of safe and affordable homes could improve health outcomes for populations, not just during times of disease outbreak (Krieger & Higgins, 2002). This can be done through a combination of strategies including permitting more apartment units and accessory dwellings (Maaoui, 2018); improving housing voucher programs and landlord uptake (HUD, 2019); maintaining and establishing emergency rental assistance to help low-income renters weather financial shocks (Hepburn et al., 2022); allocating public vacant land for affordable housing (Local Housing Solutions, 2020); tax transfer programs on sales of luxury units to fund affordable housing (Ocampo & Collins, 2022), and supporting community land trusts that steward land for affordable, quality housing (Lowery et al., 2021).

## 5 Conclusion

In this study, we found that living in substandard housing was associated with an increased risk of COVID-19 infection. The increased risk, observed only during lockdown and early reopening when residents were most exposed to their housing, suggests a causal association between poor housing and COVID-19 infection. The results demonstrate the value of combining cross-sector datasets to yield new insights and solutions. Existing city data can be leveraged to identify and prioritize 1) high-risk areas for future pandemic response activities, and 2) for longer-term solutions that address social determinants of health through safe and affordable housing.

## Financial disclosure statement

None.

## Funding

This work was supported by 10.13039/100015283Bloomberg Philanthropies.

## Ethical statement

The authors certify that this work has been completed in compliance with the Ethical Guidelines for Journal Publication. This study was reviewed and approved by the Harvard University Institutional Review Board (IRB21-0277) and the MGH Institute for Health Professions (2020P003215). The authors claim no conflict of interest and have no financial interests to disclose.

## CRediT authorship contribution statement

**Katharine Robb:** Writing – review & editing, Writing – original draft, Visualization, Supervision, Project administration, Methodology, Investigation, Formal analysis, Data curation, Conceptualization. **Rowana Ahmed:** Writing – review & editing, Validation, Methodology, Formal analysis, Data curation, Conceptualization. **John Wong:** Writing – review & editing, Data curation. **Elissa Ladd:** Writing – review & editing, Data curation. **Jorrit de Jong:** Writing – review & editing, Funding acquisition.

## Declaration of competing interest

None.

## Data Availability

The data that has been used is confidential.
