# Supplementary material for: Substandard housing and the risk of COVID-19 infection and disease severity: A retrospective cohort study
Source: SSM Popul Health. 2024 Feb 13;25:101629. doi: 10.1016/j.ssmph.2024.101629 (PMC10879830; doi:10.1016/j.ssmph.2024.101629)
Supplement: Multimedia component 1 [file mmc1.docx]

| S**upplementary Table: Characteristics of Study Population Exposed to Adequate versus Substandard Housing** | | | |  |
| --- | --- | --- | --- | --- |
|  | **Total** | **Adequate Housing, N (%)** | **Substandard Housing, N (%)** | ***p*-value** |
| **Age Distribution (N= 2873)** | | | | |
| Adults (18-64) | 2,027 (70.6) | 836 (66.9) | 1,191 (73.3) | <0.001 |
| Children (<18) | 450 (15.7) | 177 (14.2) | 273 (16.8) | 0.054 |
| Seniors (>65) | 396 (13.8) | 236 (18.9) | 160 (9.9) | <0.001 |
| **Housing/Neighborhood (N=2873)** | | | | |
| SVI Theme 1: Socioeconomic status (mean, sd) | 0.83 (0.03) | 0.82(0.04) | 0.83 (0.03) | <0.001 |
| SVI Theme 2: Household composition (mean, sd) | 0.74 (0.13) | 0.74 (0.13) | 0.74 (0.12) | 0.1744 |
| SVI Theme 3: Minority status & language (mean, sd) | 0.97 (.04) | 0.96 (0.06) | 0.98 (0.02) | <0.001 |
| SVI Theme 4: Housing type & transportation (mean, sd) | 0.84 (0.09) | 0.84 (0.08) | 0.83 (0.09) | 0.0186 |
| **COVID-19 Testing (N=2873)** | | | | |
| Lives with someone else who tested positive in study population | 585 (20.3) | 234 (18.7) | 351 (21.6) | 0.058 |
| Number of tests for COVID-19 (mean, sd) | 2.0 (2.1) | 2.2 (2.3) | 1.9 (1.9) | 0.0044 |
| **COVID-19 Severity (N=2297)** | | | | |
| Total Chronic Disease Count (mean, sd) | 2.07 (2.1) | 2.4 (2.3) | 1.8 (1.9) | <0.001 |
| Covid-related ER Visit | 119 (5.2) | 49 (4.9) | 70 (5.4) | 0.499 |
| Covid-related ICU Stay | 19 (0.8) | 7 (0.7) | 12 (0.9) | 0.541 |
| Covid-related Death | 6 (0.3) | 4 (0.4) | 2 (0.15) | 0.248 |

Note: sd=standard deviation. *P*-values result from Chi-squared test for difference in proportions or *t*-tests for difference in group means (SVI themes, number of tests for COVID-19, number of chronic diseases among those examined in table 1). SVI = The CDC/ATSDR Social Vulnerability Index, which uses 16 U.S. census variables to help local officials identify communities that may need support before, during, or after disasters (“CDC/ATSDR Social Vulnerability Index (SVI),” 2023).
